# The evaluation of the sense of hearing in patients with carotid artery stenosis within the extracranial segments

**DOI:** 10.1007/s13760-018-01058-3

**Published:** 2018-12-12

**Authors:** Karolina Dorobisz, Tadeusz Dorobisz, Dariusz Janczak, Tomasz Krecicki

**Affiliations:** Uniwersytet Medyczny im Piastow Slaskich we Wroclawiu, Wrocław, Dolnośląskie Poland

**Keywords:** Hearing, Carotid artery stenosis, Stroke, Audiological tests

## Abstract

Stenosis of arteries that supplies blood to the brain is one of the main causes of ischemic stroke which is the third most common cause of deaths in Europe. Atherosclerosis of carotid and vertebral arteries is responsible for 20% of the ischemic stroke cases. Stenosis may be either asymptomatic or manifested with typical neurological symptoms including motor and sensory disturbances as well as disturbances in vision and speech. However, discrete non-specific symptoms of ischemia, including headaches and vertigo, tinnitus and hearing loss, are also quite common. These symptoms may be indicative of a clinically significant stenosis of carotid and vertebral arteries, particularly within the internal carotid artery region, as well as of a risk of ischemic stroke. To date, research reports were unable to provide exact explanation of correlations between impaired hearing and the stenosis of carotid and vertebral arteries. Despite this, numerous articles list these symptoms as one of the first non-specific symptoms of this disorder. The ischemic mechanism within the inner ear region may lead to early symptoms of atherosclerosis of large vessels. However, no evidence of relationship and no explanation could be provided with this regard. The objective of the study was to assess the effect of carotid and vertebral artery stenosis on the function of the hearing and equilibrium organ on the basis of diagnostic audiological examinations including pure-tone threshold audiometry, impedance audiometry, otoacoustic emission tests and brainstem auditory evoked potential tests. The study was conducted in 63 patients (32 males, 31 females) aged 45–75 years, presenting with carotid and vertebral artery stenosis and treated at the Vascular Surgery Clinic of the University Clinical Hospital in Wrocław. Patients were stratified into two subgroups according to their age (45–60 years, 61–75 years). Patients were also divided into subgroups according to the stenotic arteries and to the symptomatic/asymptomatic status of the disorder. All patients were homogeneous in terms of the degree of artery stenosis. The control group consisted of 32 healthy persons (14 males, 18 females) aged 48–75 years. Patients qualified to the control group reported no history of middle or inner ear disorders, disturbed hearing, vertigo and balance disorders, as well as cardiovascular diseases; they also presented with unremarkable ultrasound scans of the arteries. All patients were subjected to precise audiological examinations. Prior to being qualified for the study, patients were subjected to the assessment of arteries by means of Doppler ultrasonography. The hearing organ function was assessed by means of pure-tone threshold audiometry, impedance audiometry, otoacoustic emission tests and brainstem auditory evoked potential tests. Reduction of the flow through the carotid arteries causes problems in the organ of hearing; abnormalities are reported especially in tone threshold audiometry, examinations of the stapedius muscle reflexes and brainstem auditory evoked potentials, which prove the presence of receptive cochlear–extracochlear hearing damage. Disturbances of the organ of hearing have similar severity in stenosis of the internal carotid artery and vertebral artery. Abnormalities found in audiologic examinations in patients with carotid artery stenosis are not always explicitly clinically expressed in patients with hearing loss; we should consider diagnostics for carotid artery stenosis.

## Introduction

Hearing disorders are a major interdisciplinary clinical problem, particularly in elderly patients. They affect about 30% of people over 65 years of age and 50% of those over 80 years of age [1]. This disease has different causes, linking the areas of knowledge from many medical specialties [1]. Otolaryngology and neurology are mainly engaged in the diagnosis and treatment of this disease. Diagnosis and treatment of patients with hearing impairments requires vast knowledge, experience and multidisciplinary diagnosis.

Carotid artery stenosis may be asymptomatic or have common neurological symptoms, such as motor and sensory disorders as well as vision or speech disturbances [2]. However, we usually encounter very discrete, non-specific symptoms of ischemia such as headaches, dizziness, tinnitus and hearing loss [3]. They can indicate clinically significant carotid artery stenosis, especially in the area of the vertebral artery and internal carotid artery, as well as suggest the risk of ischemic stroke. An overall decrease in the cerebral blood flow can also cause these symptoms. Available scientific reports do not precisely explain the correlation between hearing loss and carotid artery stenosis. Despite this, many authors mention these symptoms as one of the first and non-specific [4–7]. Diagnosis of carotid artery stenosis is based mainly on the ultrasound Duplex Doppler technique [8, 9], which is used to assess the degree of stenosis and plaque morphology, as well as classic angiography, computed tomography angiography and magnetic resonance angiography. Drug therapy or surgical treatment such as endarterectomy (the removal of plaque) or endovascular carotid stenting performed in an early period may prevent severe central nervous system complications, followed by severe disability or even death of the patient [10–12].

The literature provides many reports about significant abnormalities in audiologic and otoneurological examinations in patients with the disturbed flow in the carotid arteries [11–14]. However, the exact pathophysiological mechanism of these phenomena has not been explained. The correlation between carotid artery atherosclerosis, which significantly reduces the blood flow to the brain, and the inner ear function remains unknown. Thorough understanding of the processes taking place in the inner ear and the nervous structures of the vestibular–cochlear organ in vascular disorders can make an important contribution to the diagnosis and treatment of patients with hearing loss. Early diagnosis of these symptoms, especially unilateral, may increase detection of oligosymptomatic carotid artery stenosis and ischemic changes in the brain.

Vascular disorders of the central nervous system can cause hearing loss when they involve the labyrinthine artery. The labyrinthine artery is a branch of the anterior inferior cerebellar artery, which extends from the vertebral or basilar artery. Hearing loss occurs in 30–100% of patients with stenosis or occlusion of the labyrinthine artery [15]. Hearing disorders in the carotid vessel diseases are characterized by high volatility. They may manifest as progressive or sudden hearing loss or even sudden deafness. According to many authors, the failing function of the inner ear can appear as an isolated symptom a few days before stroke [15]. This hearing loss is sensorineural, cochlear, less often extra-cochlear, or mixed cochlear–extracochlear. Occlusion of the anterior inferior cerebellar artery is characterized by a set of symptoms typical of the failing function of the inner ear. The symptoms of sudden hearing loss are an indication for neurological diagnosis [5–7].

### Study objective

Atherosclerosis of the carotid arteries plays an important role in the aetiology of stroke, which is the third cause of death in the world, and a major factor in permanent disability in people over the age of 40 years. Ischemia of the brain affects the function of the inner ear, and thus directly hearing and balance. The disease is progressive and the symptoms worsen with the reduction of the blood flow through the carotid arteries.

Discrete hearing loss significantly affects the quality of life of the elderly, causing fear of contact with the environment and of the independent functioning. As this symptom may precede stroke, it is important to take thorough medical history, have knowledge about symptoms of the disease and implement otolaryngological–neurological diagnostics.

The aim of the study was to evaluate the effect of carotid artery stenosis on the function of hearing based on audiologic diagnosis, tone threshold audiometry, tympanometry and brainstem auditory evoked potentials.

## Material

### Control group

The control group comprised of 32 healthy patients (14 men, 18 women) aged from 48 to 75 years (mean age *M* = 61, standard deviation SD ± 7 years). The subjects classified to this group did not give a history of middle ear diseases, hearing disorders, dizziness or balance disturbances; the otolaryngological examination found no pathology; the otoscopic exam revealed normal tympanic membrane. The patients from this group have also had performed ultrasound of the carotid arteries, the result of which was normal. Medical history excluded cardiovascular diseases.

### Study group

The study comprised of 63 patients (32 men, 31 women) aged from 45 to 75 years (mean age *M* = 62.6; standard deviation SD ± 7.4 years). These were patients with carotid artery stenosis, referred to the Clinic of Vascular Surgery of the University Hospital in Wrocław in the years 2014–2015. The subjects were divided into two age groups (45–60 years, 61–75 years). The individuals excluded from the study were those with a history of cranial trauma, meningitis or neurological disorders (such as epilepsy, multiple sclerosis, Parkinson’s disease). The analysis did not take into account patients taking ototoxic drugs, subjects with a medical history eventful in terms of hearing and balance disorders, as well as those who have had any abnormalities of the organs under otolaryngological examination. During testing, none of the patients used drugs that affected the nervous system. The subjects were divided into subgroups depending on the type of narrowed artery and the presence of carotid artery stenosis symptoms. All patients constituted a homogeneous group in terms of the degree of carotid artery stenosis. The study group included patients with internal carotid artery stenosis in the range of 70–90%, or the speed of peak systolic flow in the vertebral artery above 60 cm/s.

### Method

First, detailed medical history was taken from the patients. The medical history included the underlying disease, chronic and past diseases, with particular emphasis on the pathology of the middle and inner ear and the presence of possible complications of the underlying disease, as well as conditions that may affect the sense of hearing. The risk factors of hearing damage were also assessed, i.e., exposure to noise at work or taking ototoxic medications. Each patient was consulted by a neurologist and ophthalmologist. Before inclusion of the patients in the study, the carotid arteries were evaluated using ultrasound Doppler. Each patient was subjected to an objective otolaryngological examination. Hearing was evaluated by tone threshold audiometry, tympanometry and brainstem auditory evoked potentials (BAEP).

## Results

### Analysis of data from medical history and physical examination

#### Control group

The subjects qualified to the control group did not give a history of middle or internal ear diseases, hearing loss, tinnitus, vertigo or balance disorders. The otolaryngological examination found no abnormalities. The picture of the tympanic membrane was normal in otoscopic exams in all patients from the control group. Medical history also excluded cardiovascular diseases.

#### Study group

48 (76.2%) patients from the study group gave the medical history of carotid artery stenosis symptoms. Among the ENT symptoms, unilateral hearing loss was reported by six (9.5%) patients, while bilateral hearing loss by nine subjects (14.3%).

In the group of patients with vertebral artery stenosis, unilateral hearing loss was noticed by three (13%) patients, while bilateral by one person (4.3%).

In the group of patients with internal carotid artery stenosis, unilateral hearing loss was reported by three (7.5%) and bilateral by eight (20%) patients.

### Analysis of the results of hearing examinations

In the control group, sensorineural hearing loss was found in 16 (50%) patients, whereas in the study group the same pathology occurred in 43 (68.3%) subjects.

### Analysis of the results of tone threshold audiometry

In tone audiometry, the study group demonstrated the statistically significant (*p* < 0.05) worse hearing level compared to the control group. In the left ear, the hearing threshold was higher in the study group at all frequencies; statistically significant (*p* < 0.05) differences were reported for high frequencies − 2000 Hz, 4000 Hz, 6000 Hz, 8000 Hz (Table 1).

**Table 1:** Analysis of the results of tone threshold audiometry

Tone threshold audiometry, left ear	Test group	Control group	*p*
	*N* = 63	*N* = 32	
250 Hz			
*M* ± SD	18.9 ± 10.3	15.0 ± 5.7	0.056
Me (*Q*_1_; *Q*_3_)	20 (10; 20)	13 (10; 20)	
500 Hz			
*M* ± SD	20.5 ± 9.8	17.0 ± 5.8	0.066
Me (*Q*_1_; *Q*_3_)	20 (15; 20)	18 (10; 20)	
1000 Hz			
*M* ± SD	21.7 ± 10.1	18.6 ± 6.4	0.072
Me (*Q*_1_; *Q*_3_)	20 (20; 20)	20 (15; 20)	
2000 Hz			
*M* ± SD	26.4 ± 14.6	19.7 ± 6.8	**0.013**
Me (*Q*_1_; *Q*_3_)	20 (20; 30)	20 (18; 20)	
4000 Hz			
*M* ± SD	31.6 ± 17.3	23.0 ± 11.5	**0.006**
Me (*Q*_1_; *Q*_3_)	30 (20; 40)	20 (15; 30)	
6000 Hz			
*M* ± SD	36.3 ± 19.8	24.5 ± 12.2	**0.003**
Me (*Q*_1_; *Q*_3_)	35 (20; 50)	20 (15; 35)	
8000 Hz			
*M* ± SD	38.9 ± 21.4	28.1 ± 15.3	**0.016**
Me (*Q*_1_; *Q*_3_)	40 (20; 60)	20 (18; 40)	

Bold values indicate statistically significant worse hearing level compared to the control group in left ear

In the right ear, for all tested frequencies (250 Hz, 500 Hz, 1000 Hz, 2000 Hz, 4000 Hz, 6000 Hz and 8000 Hz), the study group had significantly (*p* < 0.05) worse hearing compared with the control group (Table 2).

**Table 2:** Analysis of the results of tone threshold audiometry

Tone threshold audiometry, right ear	Test group	Control group	B vs. K *p*
	*N* = 63	*N* = 32	
250 Hz			
*M* ± SD	20.3 ± 11.4	12.5 ± 4.4	< **0.001**
Me (*Q*_1_; *Q*_3_)	20 (20; 20)	10 (10; 15)	
500 Hz			
*M* ± SD	20.0 ± 6.3	14.4 ± 5.5	< **0.001**
Me (*Q*_1_; *Q*_3_)	20 (20; 20)	10 (10; 20)	
1000 Hz			
*M* ± SD	21.4 ± 6.8	15.8 ± 5.4	< **0.001**
Me (*Q*_1_; *Q*_3_)	20 (20; 20)	18 (10; 20)	
2000 Hz			
*M* ± SD	26.1 ± 12.1	18.0 ± 6.8	< **0.001**
Me (*Q*_1_; *Q*_3_)	20 (20; 30)	20 (10; 20)	
4000 Hz			
*M* ± SD	31.7 ± 17.8	21.9 ± 8.5	**0.006**
Me (*Q*_1_; *Q*_3_)	20 (20; 40)	20 (20; 30)	
6000 Hz			
*M* ± SD	36.9 ± 19.5	26.1 ± 12.2	**0.007**
Me (*Q*_1_; *Q*_3_)	30 (20; 50)	20 (20; 35)	
8000 Hz			
*M* ± SD	40.3 ± 22.6	28.1 ± 12.3	**0.017**
Me (*Q*_1_; *Q*_3_)	40 (20; 50)	30 (20; 40)	

Bold values indicate statistically significant worse hearing level compared to the control group in right ear

The analysis of the quality of hearing in the left and right ear showed statistically significant (*p* < 0.05) differences in the study group compared with the control group. In the left ear, patients with carotid artery stenosis had significantly (*p* < 0.05) worse hearing in the high frequency bands above 4000 Hz. In the right ear, on the other hand, the significantly (*p* < 0.05) lower level of hearing quality was demonstrated in all frequencies from 250 to 8000 Hz.

### Analysis of the results of tympanometry with the examination of the stapedius muscle reflex

In all patients from the study and control group, tympanometry curves were correct—type A. However, the study group statistically significantly (*p* < 0.05) more frequently demonstrated the absence of stapedius muscle reflexes compared to the control group. The differences concerned test results both after ipsi- and contralateral stimulation. During ipsilateral stimulation, in the left ear, the stapedius muscle reflex was statistically significantly (*p* < 0.05) less frequent in all tested frequencies. In the right ear, stapedius muscle reflexes were less often recorded (58.7% vs. 75%) for the frequency of 4000 Hz, but this difference was not statistically significant. During contralateral stimulation, the examination of the stapedius muscle reflex in the study group significantly more rarely detected (*p* < 0.05) the presence of reflexes for the frequency of 2000 Hz and 4000 Hz in the right ear, but for all frequencies in both ears. In the study group, stapedius muscle reflexes were less often recorded compared with the control group, but they were not statistically significant. These data are illustrated in Tables 3 and 4.

**Table 3:** Analysis of the results of impedance audiometry with the examination of the stapedius muscle reflex ipsilateral

Stapedius reflex ipsilateral	Total *N* = 95		Test group *N* = 63		Control group *N* = 32		*B* vs. *K* *p*
	*n*	%	*n*	%	*N*	%	
500 Hz—left ear (L)	72	75.8	43	68.3	29	90.6	**0.022**
500 Hz—right ear (R)	71	74.7	47	74.6	24	75.0	0.835
L vs. R	*p* = 0.861		*p* = 0.435		*p* = 0.103		×
1000 Hz—L	70	73.7	40	63.5	30	93.8	**0.001**
1000 Hz—R	76	80.0	50	79.4	26	81.3	0.957
L vs. R	*p* = 0.305		*p* = 0.050		*p* = 0.135		×
2000 Hz—L	70	73.7	40	63.5	30	93.8	**0.001**
2000 Hz—R	67	70.5	45	71.4	22	68.8	0.974
L vs. R	*p* = 0.624		*p* = 0.346		***p*** = **0.013**		×
4000 Hz—L	64	67.4	37	58.7	27	84.4	**0.012**
4000 Hz—R	61	64.2	37	58.7	24	75.0	0.181
L vs. R	*p* = 0.642		*p* = 1.000		*p* = 0.332		×

Bold values indicate statistically significant absence of stapedius muscle reflex after ipsilateral stimulation in the study group

**Table 4:** Analysis of the results of impedance audiometry with the examination of the stapedius muscle reflex contralateral

Stapedius reflex contralateral	Razem *N* = 95		Grupa badana *N* = 63		Grupa kontrolna *N* = 32		*B* vs. *K* *p*
	*n*	%	*n*	%	*N*	%	
500 Hz—L	71	74.7	44	69.8	27	84.4	0.142
500 Hz—R	69	72.6	43	68.3	26	81.3	0.272
L vs. R	*p* = 0.743		*p* = 0.856		*p* = 0.743		×
1000 Hz—L	71	74.7	44	69.8	27	84.4	0.142
1000 Hz—R	65	68.4	39	61.9	26	81.3	0.092
L vs. R	*p* = 0.337		*p* = 0.352		*p* = 0.743		×
2000 Hz—L	61	64.2	37	58.7	24	75.0	0.181
2000 Hz—R	62	65.3	36	57.1	26	81.3	**0.035**
L vs. R	*p* = 0.874		*p* = 0.856		*p* = 0.544		×
4000 Hz—L	47	49.5	29	46.0	18	56.3	0.469
4000 Hz—R	47	49.5	25	39.7	22	68.8	**0.014**
L *vs*. R	*p* = 1.000		*p* = 0.476		*p* = 0.306		×

Bold values indicate statistically significant absence of stapedius muscle reflex after contralateral stimulation in the study group

### Analysis of the results of brainstem auditory evoked potentials

First, the results of BAEP were compared between the study and control group. Statistically significantly higher latency of the wave I, III, V and time intervals I–III, I–V, III–V were shown (*p* < 0.05) for the left and right ear in the group of patients with carotid artery stenosis as compared to the control group (Tables 5, 6). The statistically significant (*p* < 0.05) elongation of wave V was observed in the BAEP results on the right side in patients with internal carotid artery stenosis compared with the subjects with vertebral artery stenosis.

**Table 5:** Analysis of the results of brainstem auditory evoked potentials, left ear

BAEP	Test group	Control group	*p*
	*N* = 63	*N* = 32	
I [ms]			
*M* ± SD	1.92 ± 0.14	1.85 ± 0.05	< **0.001**
Me (*Q*_1_; *Q*_3_)	1.93 (1.87; 1.98)	1.86 (1.82; 1.88)	
III [ms]			
*M* ± SD	4.35 ± 0.25	4.19 ± 0.10	< **0.001**
Me (*Q*_1_; *Q*_3_)	4.31 (4.20; 4.50)	4.18 (4.12; 4.25)	
V [ms]			
*M* ± SD	5.90 ± 0.24	6.00 ± 0.10	**0.023**
Me (*Q*_1_; *Q*_3_)	5.91 (5.72; 6.02)	5.99 (5.94; 6.04)	
I–III			
*M* ± SD	2.44 ± 0.21	2.34 ± 0.09	**0.0002**
Me (*Q*_1_; *Q*_3_)	2.43 (2.34; 2.52)	2.35 (2.27; 2.40)	
I–V			
*M* ± SD	4.00 ± 0.21	4.14 ± 0.08	< **0.001**
Me (*Q*_1_; *Q*_3_)	4.01 (3.82; 4.13)	4.14 (4.09; 4.18)	
III–V			
*M* ± SD	1.56 ± 0.19	1.80 ± 0.08	< **0.001**
Me (*Q*_1_; *Q*_3_)	1.54 (1.41; 1.73)	1.81 (1.76; 1.85)	

Bold values indicate statistically significant higher latency of waves in BAEP in the study group in left ear

**Table 6:** Analysis of the results of brainstem auditory evoked potentials, right ear

BAEP	Test group	Control group	p
	*N* = 63	*N* = 32	
I [ms]			
*M* ± SD	1.91 ± 0.12	1.86 ± 0.06	**0.003**
Me (*Q*_1_; *Q*_3_)	1.91 (1.85; 1.98)	1.86 (1.84; 1.89)	
III [ms]			
*M* ± SD	4.36 ± 0.26	4.22 ± 0.16	**0.003**
Me (*Q*_1_; *Q*_3_)	4.35 (4.18; 4.51)	4.20 (4.14; 4.25)	
V [ms]			
*M* ± SD	5.92 ± 0.26	5.98 ± 0.09	**0.023**
Me (*Q*_1_; *Q*_3_)	5.89 (5.72; 6.06)	5.96 (5.94; 6.00)	
I–III			
*M* ± SD	2.45 ± 0.23	2.37 ± 0.17	**0.008**
Me (*Q*_1_; *Q*_3_)	2.46 (2.34; 2.63)	2.32 (2.28; 2.40)	
I–V			
*M* ± SD	3.96 ± 0.45	4.13 ± 0.09	< **0.001**
Me (*Q*_1_; *Q*_3_)	3.97 (3.80; 4.15)	4.12 (4.08; 4.16)	
III–V			
*M* ± SD	1.56 ± 0.24	1.76 ± 0.16	< **0.001**
Me (*Q*_1_; *Q*_3_)	1.53 (1.40; 1.71)	1.79 (1.72; 1.84)	

Bold values indicate statistically significant higher latency of waves in BAEP in the study group in right ear

Next, the analysis was conducted to compare the number of normal and abnormal results in the study group compared with the control group (Table 7). The patients with carotid artery stenosis more often demonstrated abnormal results, especially the latency of wave I was significantly (*p* < 0.05) more frequently incorrect on the right and left sides compared to the results of patients from the control group. Abnormal parameters in the latency of wave V and time interval I–III more often appeared on the left side, while in time interval I–V on the right side, although the results showed no statistically significant differences.

**Table 7:** Analysis of the results of brainstem auditory evoked potentials, analysis of the number of normal and abnormal results in the study group compared with the control group

	Test group *N* = 63		Control group *N* = 32		*p*
	*N*	%	*N*	%	
Left ear I latency					
< 1.9 ms	27	42.9	27	84.4	< 0.001
≥ 1.9 ms	36	57.1	5	15.6	
Left ear V latency					
< 6.2 ms	58	92.1	31	96.9	0.660
≥ 6.2 ms	5	7.9	1	3.1	
Left ear I–III interpeak latency					
< 2.6 ms	52	82.5	31	96.9	0.055
≥ 2.6 ms	11	17.5	1	3.1	
Left ear III–V Interpeak latency					
< 2.4 ms	63	100.0	32	100.0	1.000
≥ 2.4 ms	0	0.0	0	0.0	
Left ear I–V interpeak latency					
< 4.6 ms	63	100.0	32	100.0	1.000
≥ 4.6 ms	0	0.0	0	0.0	
Right ear I latency					
< 1.9 ms	29	46.0	27	84.4	< 0.001
≥ 1.9 ms	34	54.0	5	15.6	
Right ear V latency					
< 6.2 ms	55	87.3	31	96.9	0.265
≥ 6.2 ms	8	12.7	1	3.1	
Right ear I–III interpeak latency					
< 2.6 ms	46	73.0	29	90.6	0.062
≥ 2.6 ms	17	27.0	3	9.4	
Right ear III–V interpeak latency					
< 2.4 ms	63	100.0	32	100.0	1.000
≥ 2.4 ms	0	0.0	0	0.0	
Right ear I–V interpeak latency					
< 4.6 ms	61	96.8	32	100.0	0.548
≥ 4.6 ms	2	3.2	0	0.0	

## Discussion

In elderly patients, diseases of the inner ear are very often overlooked in the diagnosis, both by patients and physicians. In most cases, hearing loss is caused by harmless factors. Even though the patient experiences a reduction in the quality of life, there is no risk of complications leading to further deterioration of health or death.

The aging of the organ of Corti is the most common cause of hearing loss in the elderly [5, 7]. Hearing loss may be the only preceding symptom of stroke [4, 6], but the impact of flow disturbances in the carotid arteries on the inner ear has not yet been thoroughly investigated.

The technique of audiologic and otoneurological tests is also a challenge in elderly people, especially those with dementia. We also need to improve public awareness and introduce appropriate prophylaxis in this regard.

All subjects were subjected to audiologic examinations. Tone threshold audiometry, tympanometry with the assessment of the stapedius muscle reflex, brainstem auditory evoked potentials are the best diagnostic tools for the evaluation of hearing loss causes. Lin et al. [16] and Roth et al. [17] point out that tone threshold audiometry is particularly important as screening and should be implemented in the elderly. Its result ought to be a basis for a decision to refer the patient to further diagnostics, and not just hearing prosthesis. The evaluation of brainstem auditory evoked potentials is a very important part of diagnostics allowing for the identification of the cause of extra-cochlear hearing loss or mixed damage to the cochlea. It also allows for the tracking of the auditory pathway and thus sensibly directs the attention to central nervous system disorders and their frequent ischemic background.

Comparative analysis of hearing in the control group found hearing loss in 50% of patients as compared to 68.3% of subjects from the study group.

In tone threshold audiometry, statistically significant (*p* < 0.05) worse hearing was remarkable in patients from the study group at high frequencies 2000–8000Hz in the left ear and at all tested frequencies from 250 to 8000 Hz in the right ear.

Many literature reports have noted that hearing loss is often accompanied by neurological disorders and cerebral ischemia [7]. Ravecca et al. [18] and Yin et al. [19] indicated in their publications that the symptom of sudden deafness by a few days preceded ischemic stroke.

After dividing the patients into subgroups based on the level of hearing quality (normal hearing, mild, moderate, severe hearing loss and hearing remnants), statistically significant (*p* < 0.05) differences were reported in the left ear for the frequency above 4000 Hz and in the right ear for all tested frequencies. The patients with carotid arteries stenosis were much more likely to have moderate or severe hearing loss compared to the control group. Morl et al. [4] and Bohme [5] confirm the increased incidence of hearing loss in patients with carotid artery stenosis.

In tympanometry with the assessment of the stapedius muscle reflexes, all patients from the control and study group demonstrated normal movement of the tympanic membrane—tympanometry curves type A, however, in the study group, the stapedius muscle reflexes were registered statistically significantly less often (*p* < 0.05). According to other authors, in patients with carotid artery stenosis, damage or impairment of nerve conduction may be the first prodromal symptom of stroke [7]. The function of the stapedial nerve (a branch of the facial nerve) is possible to evaluate in a simple and non-invasive test, which takes a few seconds. Taking into account study results and the fact that the available literature lacks discussion about stapedius muscle reflexes disturbances in patients with carotid artery stenosis further discussion on this subject is necessary.

Comparative analysis of the BAEP results showed the statistically significant (*p* < 0.05) elongation of wave I, III, V and time intervals I–III, I–V and III–V in patients with carotid artery stenosis compared to the subjects from the control group.

The patients with vertebral artery stenosis significantly (*p* < 0.05) more frequently demonstrated the elongation of wave V compared to patients with internal carotid artery stenosis.

The subjects from the study group with neurological symptoms also had prolonged latency of waves I, III, V and time intervals I–II and I–V.

The analysis of BAEP results in terms of the number of abnormal results revealed the elongation of wave I in about 50% of patients with vertebral artery stenosis and in approximately 60% of those with internal carotid artery stenosis. On the other hand, less than 10% of patients with stenosis of the vertebral artery and internal carotid artery showed the elongation of wave V. In patients from the study group with neurological symptoms, the elongation of wave I latency was recorded in 72% for the left ear and in 60% for the right ear, while in patients with otolaryngological symptoms, this phenomenon occurred in approximately 60%. Interestingly, among patients without neurological symptoms, more than 50% revealed the elongation of wave I latency, which proved the presence of a subclinical disorder. Similar observations concern asymptomatic patients without ENT symptoms, where 44.4% displayed the elongation of wave I latency. The BAEP results showed a significant delay in the auditory pathway conduction in patients with carotid artery stenosis compared to the control patients. Patients from the study group, also those asymptomatic, had abnormalities in response to auditory evoked potentials, indicating subclinical damage to the auditory pathway. This also proves BAEP to be an important element of clinical diagnosis in suspected hearing loss, and points to the need to refer patients to further diagnosis for flow disturbances in carotid arteries or other neurological disorders. In many reports [16–19], authors suggest that BAEP abnormalities oblige to perform further imaging diagnostics of the brain to rule out neurological diseases. However, I have never encountered a study that would emphasise the need for ultrasound diagnostics of the carotid artery flow in patients with abnormal BAEP results. This fact requires further observation, as well as directing the attention of ENTs to the problem of carotid artery stenosis in patients with hearing loss and the necessity to perform a detailed examination of the flow in the carotid arteries in these patients.

## Conclusions

1. Reduction of the flow through the carotid arteries causes problems in the organ of hearing; abnormalities are reported especially in tone threshold audiometry, examinations of the stapedius muscle reflexes and BAEP, which proves the presence of sensorineural cochlear–extracochlear hearing damage.
2. Disturbances of the organ of hearing have similar severity in stenosis of the internal carotid artery and vertebral artery.
3. Abnormalities found in audiologic examinations in patients with carotid artery stenosis are not always explicitly clinically expressed.
4. In patients with hearing loss, we should consider diagnostics for carotid artery stenosis.
